# Sentinel Lymph Node Biopsy in Low-Risk Breast Cancer in Patients ≥ 70 Years: How Wisely are We Choosing?

**DOI:** 10.1245/s10434-025-18534-9

**Published:** 2025-10-30

**Authors:** Gabriella N. Tortorello, Neha Shafique, Elizabeth M. De Jesus, Phyllis Gimotty, Anushka Dheer, Oluwadamilola M. Fayanju, Julia Tchou, John T. Miura, Giorgos C. Karakousis, Katharine Rendle

**Affiliations:** 1 Department of Surgery, Hospital of the University of Pennsylvania, Philadelphia, PA USA; 2 Department of Biostatistics, Epidemiology, and Bioinformatics, Perelman School of Medicine, University of Pennsylvania, Philadelphia, USA; 3 Department of Family Medicine and Community Health, Perelman School of Medicine, University of Pennsylvania, Philadelphia, USA

**Keywords:** Breast cancer, De-escalation of care, Sentinel lymph node, Sentinel lymph node biopsy

## Abstract

**Introduction:**

The Choosing Wisely (CW) guidelines in 2016 recommended against routine sentinel lymph node biopsy (SLNB) for women 70 years and older with early-stage, low-risk breast cancer. We sought to examine trends in SLNB before and after CW guidelines along with the subsequent impact on adjuvant therapy.

**Methods:**

The National Cancer Database was used to identify women aged 70 years and older with clinical stage I, ER/PR+, HER2− breast cancer between 2010 and 2021. We evaluated annual percent change (APC) in rates of SLNB by Joinpoint log-linear regression and used a multivariable logistic regression model to identify predictors of receipt of SLNB in the post-CW cohort. We also assessed rates of adjuvant chemotherapy and radiation stratified by receipt of SLNB in the post-CW cohort.

**Results:**

Our study demonstrated an annual decrease in the percentage of women undergoing SLNB after 2016, with an APC of −4.1% (*p* < 0.001); however, a majority of patients meeting de-escalation criteria (68.7%) still underwent SLNB in 2021. Women older than 75 (OR 0.31, 95% CI 0.30–0.32) and with a Charlson–Deyo score of 3 or greater (OR 0.65, 95% CI 0.60–0.70) were least likely to undergo SLNB, while treatment at nonacademic centers was associated with SLNB (OR 1.70, 95% CI 1.63–1.76). Post-CW, there were no significant change in rates of adjuvant chemotherapy. However, receipt of adjuvant radiation therapy significantly increased in patients for whom SLNB was omitted with an APC of +15.0%, with each additional year of diagnosis after 2016 associated with increased odds of radiation receipt (OR 1.14, 95% CI 1.11–1.17).

**Conclusions:**

Though there has been significant progress made in de-escalating surgical management of early-stage breast cancer in older patients, most patients are still undergoing SLNB. Moreover, patients in whom SLNB is being omitted appear more likely than before to receive adjuvant radiation. More work is needed to provide quality, uniform and cost-effective care for all patients.

In its 2016 Choosing Wisely (CW) guidelines, the Society of Surgical Oncology recommended against routine sentinel lymph node biopsy (SLNB) for women 70 years and older with early-stage, low-risk breast cancer on the basis of a strong body of evidence that axillary staging in this cohort does not impact mortality or local recurrence.^1–5^ In this population, omitting this procedure may help decrease operative morbidity as well as minimize the risk of unnecessary, burdensome treatment. Prior literature has shown that despite the guidelines, more than 80% of patients who meet de-escalation criteria for omission of SLNB continued to undergo the procedure.^6–10^ Still, little is known about how these guidelines have impacted longitudinal trends in application of SLNB in older women with low-risk breast cancer within a national cohort. Our primary aim was to explore trends in SLNB before and after the publication of the CW guidelines. Furthermore, we sought to examine the impact of the CW guidelines on adjuvant therapies. Previous work has demonstrated that SLN positivity in the patient population has a strong association with receiving adjuvant radiation therapy, as may be receipt of the procedure itself.^6,8^ In our study, we specifically aimed to explore trends in postoperative radiation and chemotherapy with a particular focus on patients in whom SLNB is omitted.

## Patients and Methods

We performed a retrospective cohort study using the National Cancer Database (NCDB), which captures more than 70% of new cancer diagnoses in the USA on an annual basis. We selected women who would otherwise meet criteria for omission of SLNB according to the CW guidelines. Specifically, the study included women aged 70 years and older with American Joint Committee on Cancer (AJCC) 8 clinical stage I, ER/PR+, HER2− breast cancer diagnosed between 2010 and 2021. Accordingly, all patients had tumors 2 cm and smaller and had no evidence of clinical nodal disease or distant metastases. All patients underwent surgical excision of their primary tumor, and we excluded anyone who underwent neoadjuvant therapy. We also excluded patients with missing data pertaining to SLNB status and/or lymph node positivity status.

To examine trends in SLNB, we first calculated the percentage of patients meeting our inclusion criteria who underwent the procedure during each year of the study and then evaluated the significance of the annual percent change by Joinpoint log-linear regression. Overall characteristics of our cohort were explored with nonparametric descriptive statistics, specifically percentages, medians, and interquartile ranges. To identify factors associated with continued application of SLNB after publication of the CW guidelines (post-CW), we then focused on patients diagnosed from 2017 to 2021 (post-CW cohort). We examined the distribution of baseline characteristics across the two treatment groups in the post-CW cohort—SLNB and no-SLNB—using Pearson’s chi-squared and Wilcoxon rank sum tests for categorical and continuous variables, respectively. We then built a multivariable logistic regression model to better assess the impact of patient-, disease-, and treatment-related factors on receipt of SLNB in the post-CW era. We included factors determined a priori to be related to receipt of SLNB as well as those found to be unevenly distributed across groups on univariable analysis, and we performed backwards stepwise selection to select our final model. We did not include lymphovascular invasion (LVI) in our model of receipt of biopsy because in the NCDB, LVI data is populated from either core biopsy or surgical pathology, and we were most interested in exploring factors present preoperatively that may have informed decision-making. We also examined predictors of nodal positivity post-CW, again using both univariable analysis as well as logistic regression modeling.

To explore the impact of receipt of SLNB on future treatment in the post-CW cohort, we compared rates of adjuvant chemotherapy and radiation between SLNB and no-SLNB groups. Adjuvant radiation was inclusive of radiation delivered to the breast and/or the axilla. In patients who underwent SLNB, adjuvant radiation was defined as radiation received after SLNB and was stratified by result of biopsy. In patients in whom SLNB was omitted, adjuvant radiation was defined as radiation received after surgical excision of their primary tumor. We used logistic regression to explore the impact of SLNB on the receipt of these additional therapies adjusted for confounding. A *p*-value of < 0.05 was considered statistically significant throughout. Trend analyses were conducted using National Cancer Institute Joinpoint Statistical Software, version 5.0. All other analyses were performed using Stata statistical software, version 17.0.

## Results

### Demographics of the Cohort

A total of 162,975 women met inclusion criteria. The median age was 75 years (interquartile range [IQR] 72–80). Exactly 88% (*n* = 139,686) of women were non-Hispanic White, and 6% were non-Hispanic Black. Most patients (75%) were treated at nonacademic medical centers, and most underwent breast-conserving surgery (83%) rather than mastectomy. Table 1 shows the baseline characteristics of the cohort.

**Table 1:** Demographics and clinical characteristics of the study population

	Total *n* (%)	No SLNB *n* (%)	SLNB *n* (%)
Total subjects	162,975	32,929	130,046
Age, median (IQR)	75 (72–80)	80 (75–85)	75 (72–79)
Race/ethnicity			
Non-Hispanic White	139,686 (88.0)	22,298 (88.1)	111,399 (87.9)
Non-Hispanic Black	9718 (6.1)	1996 (6.2)	7722 (6.1)
Hispanic/Latino	4901 (3.1)	856 (2.7)	4045 (3.2)
AAPI	4086 (2.6)	837 (2.6)	3249 (2.6)
Unknown	460 (0.3)	115 (0.4)	345 (0.3)
CDCC			
0	122,559 (75.2)	23,898 (72.6)	98,661 (75.9)
1	27,705 (17.0)	5620 (17.1)	22,085 (17.0)
2	8048 (4.9)	2001 (6.1)	6047 (4.6)
3+	4663 (2.9)	1410 (4.3)	3253 (2.5)
Insurance status			
Medicare	143,600 (88.1)	29,328 (89.1)	114, 272 (87.9)
Private	15,274 (9.4)	2775 (8.4)	12,499 (9.6)
Medicaid	1733 (1.1)	352 (1.1)	1381 (1.1)
Unknown	1986 (1.2)	402 (1.2)	1,84 (1.2)
Uninsured	382 (0.2)	72 (0.2)	310 (0.2)
Hospital status			
Nonacademic	121,659 (74.7)	22,298 (67.7)	99,361 (76.4)
Academic	41,316 (25.3)	10,631 (32.3)	30,685 (23.6)
Hospital setting			
Metropolitan	139,463 (87.2)	29,061 (90.2)	110,402 (86.5)
Urban	18,136 (11.3)	2859 (8.9)	15,277 (12.0)
Rural	2314 (1.5)	304 (0.9)	2010 (1.6)
Tumor T-stage			
T1a	20,122 (12.4)	4862 (14.8)	15,260 (11.7)
T1b	59,542 (36.5)	12,518 (38.0)	47,024 (36.2)
T1c	83,311 (51.1)	15,549 (47.2)	67,762 (52.1)
Breast surgery type			
Lumpectomy	135,452 (83.1)	29,207 (88.7)	106,245 (81.7)
Mastectomy, no recon	23,100 (14.2)	3191 (9.7)	19,909 (15.3)
Mastectomy, with recon	4293 (2.6)	417 (1.3)	3876 (3.0)
Unknown	129 (0.1)	114 (0.3)	15 (< 0.1)

### Trends in SLNB

**Fig. 1:**
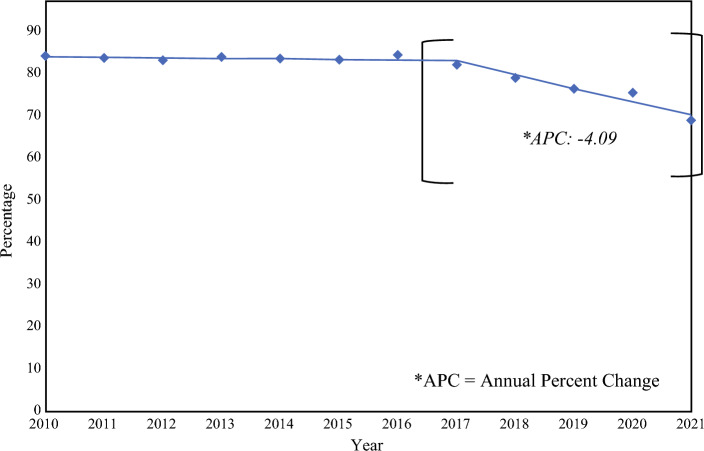
Percentage of patients undergoing sentinel lymph node biopsy (SLNB) despite meeting de-escalation criteria

Between 2010 and 2015, prior to the publication of the CW-guidelines (pre-CW), 83.5% of patients underwent SLNB. Post-CW, we observed an annual decrease in the percentage of women meeting de-escalation criteria undergoing SLNB, with an annual percent change of 4.1% using log linear regression (*p* < 0.001), Fig. 1. Overall, 75.9% of women underwent SLNB post-CW, with this number decreasing from 81.6% in 2017 to 68.7% in 2021.

### Predictors of Receipt of SLNB

Table 2 presents the distribution of patient and clinical characteristics across the SLNB and no-SLNB treatment groups in the post-CW era. On univariable analysis, we found that patients undergoing SLNB were younger (median age 74 versus 78, *p* < 0.001) and had fewer medical comorbidities (91% CDCC 0-1 versus 88%, *p* < 0.001). In addition, patients undergoing SLNB were more likely to be treated at nonacademic medical centers (76% versus 67%, *p* < 0.001) and in nonmetropolitan settings (14% versus 10%, *p* < 0.001). A greater percentage of patients who underwent SLNB were diagnosed with T1c tumors rather than T1a–b (53% versus 46%, *p* < 0.001), and a greater percentage underwent mastectomy rather than breast-conserving surgery (16.8% versus 8.1%, *p* < 0.001).

**Table 2:** Characteristics of the post-CW cohort by SLNB status

	SLNB *n* (%)	No SLNB *n* (%)	*p*-Value
Total subjects	62,827 (75.9)	19,928 (24.1)	
Age			
70–75	37,615 (85.4)	6452 (14.6)	< 0.001
< 75	25,212 (65.2)	13,476 (34.8)	
Race/ethnicity			
Non-Hispanic White	53,718 (75.9)	17,054 (24.1)	< 0.001
Non-Hispanic Black	3883 (76.1)	1220 (23.9)	
Hispanic/Latino	2140 (78.9)	572 (21.1)	
AAPI	1701 (74.8)	574 (25.2)	
Unknown	173 (70.6)	72 (29.5)	
CDCC score			
0	47,383 (76.8)	14,350 (23.2)	< 0.001
1	9863 (75.6)	3192 (24.4)	
2	3283 (71.8)	1288 (28.2)	
3+	2298 (67.7)	1098 (32.3)	
Insurance status			
Medicare	55,729 (75.8)	17,819 (24.2)	0.046
Private	5502 (77.3)	1615 (22.7)	
Medicaid	702 (77.4)	205 (22.6)	
Unknown	766 (75.8)	245 (24.2)	
Uninsured	128 (74.4)	55 (25.6)	
Hospital status			
Non-academic	48,009 (78.3)	13,277 (21.7)	< 0.001
Academic	14,818 (69.0)	6651 (31.0)	
Hospital setting			
Metropolitan	53,179 (75.2)	17,562 (24.8)	< 0.001
Urban	7648 (81.3)	1757 (18.7)	
Rural	966 (84.3)	180 (15.7)	
Tumor T-stage			
T1a	7465 (71.1)	3034 (28.9)	< 0.001
T1b	22,150 (74.2)	7700 (25.8)	
T1c	33,212 (78.3)	9194 (21/7)	
Breast surgery type			
Lumpectomy	18,238 (25.8)	52,471 (74.2)	< 0.001
Mastectomy, no recon	1389 (14.3)	8324 (85.7)	
Mastectomy, with recon	222 (9.8)	2038 (90.2)	

Table 3 presents the results of our multivariable logistic regression model examining factors associated with receipt of SLNB post-CW. The results were overall consistent with our unadjusted analysis though insurance status was no longer associated with likelihood of receiving SLNB. We found that after adjusting for all other factors, women who were older than 75 were less likely to undergo node biopsy compared with those aged 70–75 (OR 0.31, 95% CI 0.30–0.32). Women with a CDCC score of 3 or greater had 35% decreased odds of SLNB compared with women with no medical comorbidities (OR 0.65, 95% CI 0.60–0.70). As in our univariable analysis, treatment at nonacademic centers was associated with SLNB after adjusting for other factors (OR 1.70, 95% CI 1.63–1.76), as was treatment in nonmetropolitan settings. Patients with higher clinical T-stage disease had greater odds of SLNB (T1c versus T1a OR 1.52, 95% CI 1.44–1.60), as did patients who underwent mastectomy either with or without reconstruction (OR 2.13, 95% CI 2.00–2.27).

**Table 3:** Factors associated with SLNB in the post-CW era as predicted by multivariable logistic regression, *n* = 82,755

	OR	95% CI	*p*-Value
Age			
70–75	1 (reference)		
> 75	0.31	0.30–0.32	< 0.001
Race/ethnicity			
Non-Hispanic White	1 (reference)		
Non-Hispanic Black	1.09	1.01–1.17	0.022
Hispanic/Latino	1.23	1.11–1.36	< 0.001
AAPI	0.90	0.81–0.99	0.046
Unknown	0.78	0.58–1.06	0.11
CDCC score			
0	1 (reference)		
1	0.91	0.86–0.95	< 0.001
2	0.77	0.72–0.83	< 0.001
3+	0.65	0.60–0.70	< 0.001
Insurance status			
Medicare	1 (reference)		
Private	0.96	0.90–1.02	0.19
Medicaid	0.97	0.82–1.15	0.75
Unknown	0.98	0.84–1.14	0.78
Uninsured	0.90	0.62–1.31	0.58
Hospital status			
Academic	1 (reference)		
Non-academic	1.70	1.63–1.76	< 0.001
Hospital setting			
Metropolitan	1 (reference)		
Urban	1.39	1.31–1.47	< 0.001
Rural	1.75	1.48–2.07	< 0.001
Tumor T-stage			
T1a	1 (reference)		
T1b	1.20	1.14–1.26	< 0.001
T1c	1.53	1.45–1.61	< 0.001
Breast surgery type			
Lumpectomy	1 (reference)		
Mastectomy, no recon	2.10	1.97–2.24	< 0.001
Mastectomy, with recon	2.41	2.08–2.79	< 0.001

We a performed a subgroup analysis of patients undergoing lumpectomy only given the possibility of surgeon concern about the lack of future ability to track the lymphatic drainage following mastectomy. The results of this logistic regression model aligned with that modelling our entire cohort and are shown in Table 4. Overall, 73.8% of patients undergoing lumpectomy also underwent SLNB after 2016, compared with 75.6% of the entire cohort.

**Table 4:** Factors associated with SLNB in the post-CW era as predicted by multivariable logistic regression, lumpectomy patients only, *n*=70,709

	OR	95% CI	*p*-Value
Age			
70–75	1 (reference)		
> 75	0.29	0.28–0.30	< 0.001
Race/ethnicity			
Non-Hispanic White	1 (reference)		
Non-Hispanic Black	1.04	0.97–1.13	0.25
Hispanic/Latino	1.22	1.10–1.35	< 0.001
AAPI	0.84	0.75–0.93	0.002
Unknown	0.80	0.58–1.11	0.19
CDCC score			
0	1 (reference)		
1	0.90	0.85–0.94	< 0.001
2	0.73	0.68–0.79	< 0.001
3+	0.62	0.57–0.67	< 0.001
Insurance status			
Medicare	1 (reference)		
Private	0.97	0.90–1.03	0.34
Medicaid	0.97	0.81–1.16	0.75
Unknown	0.95	0.81–1.12	0.55
Uninsured	0.82	0.55–1.23	0.34
Hospital status			
Academic	1 (reference)		
Nonacademic	1.77	1.70–1.84	< 0.001
Hospital setting			
Metropolitan	1 (reference)		
Urban	1.39	1.30–1.47	< 0.001
Rural	1.76	1.47–2.11	< 0.001
Tumor T-stage			
T1a	1 (reference)		
T1b	1.22	1.15–1.29	< 0.001
T1c	1.56	1.48–1.65	< 0.001

### Predictors of Nodal Positivity

We then explored rates and predictors of nodal positivity amongst those who continued to undergo SLNB after 2016, Table 5. We did find that after the new guidelines, there was a small but significant increase in the percentage of patients with a positive sentinel node (post-CW 7.6% versus pre-CW 6.6%, *p* < 0.001). Patients whose tumors showed evidence of LVI on either core biopsy or final surgical pathology had greater than five-fold increased odds of having a positive SLN (OR 6.46, 95% CI 5.95–7.01), and amongst patients receiving SLNB, 32.3% of patients with tumors showing LVI had a positive node. Overall, 5.4% of tumors showed LVI post-CW, regardless of SLNB status. AJCC clinical T stage also had a significant association with nodal positivity. Exactly 11% of patients with T1c tumors undergoing SLNB had a positive node compared with 1.6% of T1a and 4.5% of T1b tumors (*p* < 0.001). After adjusting for patient and disease factors, having a T1c tumor was associated with six times the odds of having a positive node (OR 6.11, 95% CI 5.07–7.35).

**Table 5:** Factors associated with nodal positivity in the post-CW era as predicted by multivariable logistic regression, *n* = 62,827

	% with a positive node	OR	95% CI	*p*-Value
Age				
70–75	7.5	1 (reference)		
> 75	7.8	0.99	0.93–1.06	0.85
Race/ethnicity				
Non-Hispanic White	7.5	1 (reference)		
Non-Hispanic Black	9.3	1.17	1.04–1.32	0.008
Hispanic/Latino	7.7	0.93	0.79–1.10	0.41
AAPI	7.6	0.92	0.76–1.12	0.41
Unknown	3.5	0.42	0.18–0.97	0.043
CDCC score				
0	7.5	1 (reference)		
1	7.5	0.97	0.89–1.06	0.52
2	8.7	1.10	0.96–1.26	0.16
3+	9.6	1.22	1.05–1.42	0.01
Insurance status				
Medicare	7.6	1 (reference)		
Private	7.8	1.07	0.96–1.19	0.23
Medicaid	8.1	1.01	0.76–1.35	0.93
Unknown	8.9	1.25	0.96–1.64	0.10
Uninsured	10.9	1.49	0.83–2.67	0.18
Tumor T-stage				
T1a	1.7	1 (reference)		
T1b	4.4	2.59	2.14–3.13	< 0.001
T1c	11.1	6.03	5.02–7.23	< 0.001
Lymphovascular invasion				
Absent	5.7	1 (reference)		
Present	32.4	6.49	5.99–7.04	< 0.001
Unknown	9.5	1.75	1.60–1.93	< 0.003

### Changes in Postoperative Therapies

**Fig. 2:**
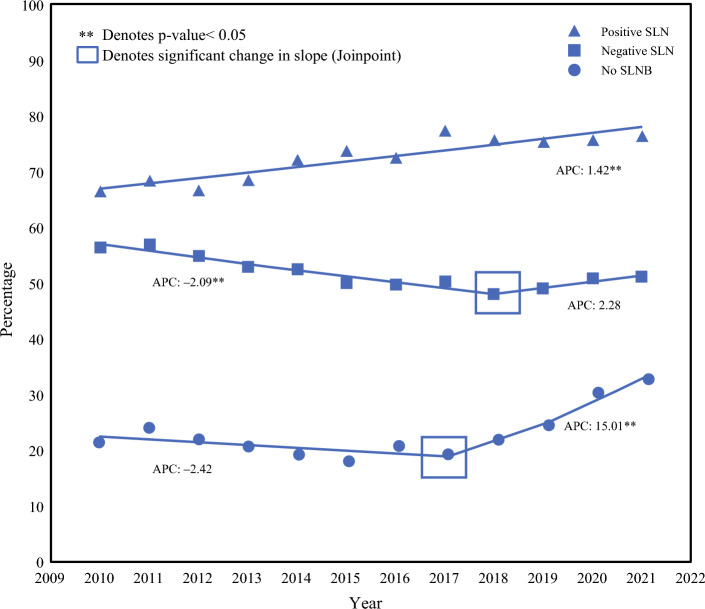
Percentage of patients receiving postoperative radiation therapy with annual percent change (APC) by Joinpoint analysis, stratified by sentinel lymph node biopsy (SLNB) and positivity status

Pre-CW, 49.1% of patients underwent postoperative radiation therapy compared with 45.7% of patients post-CW. Figure 2 shows the annual percentages of patients receiving radiation by sentinel lymph node status. Most notably, for patients for whom SLNB was omitted in concordance with CW guidelines, there was a significant increase in the receipt of adjuvant radiation therapy post-CW, with an annual change of 15.0%. For patients with a positive SLN, the rate of adjuvant radiation increased gradually over the interval, with an annual change of 1.42% (*p* < 0.01). For patients with a negative SLN, there was a small decrease in the percentage of patients undergoing adjuvant radiation pre-CW, with a flattening of the rate beginning in 2018. These same trends persisted on subgroup analysis examining only patients who received breast-conserving surgery. In addition, in a multivariable logistic regression model predicting the likelihood of adjuvant radiation amongst patients who did not undergo SLNB, each additional year of diagnosis after 2016 was associated with 14% increased odds of radiation receipt (OR 1.14, 95% CI 1.11–1.17).

Overall, 3.2% of patients underwent adjuvant chemotherapy pre-CW compared with 3.4% post-CW (*p* = 0.18). Furthermore, there was no significant change in the annual percentage of adjuvant chemotherapy in this cohort overall or on stratified analysis by sentinel lymph node status (negative, positive, or not sampled).

## Discussion

Overall, we found that there has been an annual decrease in the percentage of women undergoing SLNB following release of the CW guidelines, although the majority of women (68.7%) who meet guidelines are still undergoing SLNB in most recent year evaluable (2021). These national data are more current than those previously published.^7,11^ While other authors have similarly examined adherence to the CW guidelines, our study focuses on national and longitudinal trends of guideline adherence before and after the guidelines were published as well as factors associated with compliance to guidelines and implications on adjuvant treatments for patients no longer undergoing SLNB. Patients least likely to undergo SLNB in the post-CW era were found to be older, more medically complex, and more likely to be treated at metropolitan, academic medical centers. Interestingly, there was a slight increase in SLN positivity rates among patients undergoing SLNB in the post-CW era suggesting improved selection of patients for the procedure, although rates overall remained less than 10%. The greatest predictors of SLN positivity post-CW guidelines were LVI and clinical T-stage, which is concordant with previous work.^12–14^

De-escalation of care matters because while SLNB is generally a very safe procedure, it is not without risk. Rates of lymphedema have been reported to be between 3.7 and 7.5%.^15^ There is also a risk of upper-limb morbidity with a 17% risk of reduced range of motion and 15% risk of reduced strength.^15^ In addition, studies have shown that there is discordance between who among patients with early-stage breast cancer is most at risk for recurrence and who is most likely to receive SLNB.^16^ The risks of the procedure may be particularly unacceptable among these patients. While some insitutions and providers are able to perform both breast surgery and nodal sampling under sedation, SLNB is also typically more likely to require general anesthesia and the risks therein than a lumpectomy or mastectomy alone. We were interested to see if there was any abrupt change in the rate of SLNB in 2020 associated with the coronavrisu disease 19 (COVID-19) pandemic, and we had hypothesized that there would be a more significant decrease in the percentage of patients undergoing SLNB that year as to not place patients or providers at risk of disease transmission in the peri-anesthesia period. We did not find this to be true, however, and from 2019 to 2020 rates of SLNB remained relatively flat, which may reflect an attempt to consolidate all care into as few encounters as possible given the instability of the healthcare system that year.

Prior qualitative work has examined some of the factors limiting guideline implementation.^7,17,18^ Some components can simply be attributed to lack of provider awareness of the CW guidelines, and the improved adherence we observed may be explained by the dissemination of information over time. There is also reluctance by patients to forgo a procedure that is perceived of as being very low-risk, providing “peace of mind,” and potentially informative regarding options for adjuvant therapy.^19^ This is particularly important when there is a lack of buy-in from the interdisciplinary care team.^18^ Both patients and providers can also feel that functional status is more important than chronologic age and therefore think that the CW guidelines only apply to older women in relatively poor health.^18,19^ Positive reframing and emphasizing the good prognosis that undergirds the CW guidelines may help address concerns about disease progression and age discrimination expressed by patients.^19^

The recent SOUND trial explored the omission of SLNB in low-risk breast cancer regardless of age after negative clinical axillary staging (ultrasound and physical exam), theoretically allowing for recommendations similar to CW to be applicable to younger patients. The SOUND trial found no differences in rates of recurrence or adjuvant therapies between patients who did or did not undergo SLNB.^20^ Clinical guidelines have not yet changed to reflect these findings. Importantly, the SOUND trial predates the recent promising findings of decreased recurrence with cyclin-dependent kinase (CDK) inhibitors for high-risk early breast cancer in recent clinical trials which may further omission of SLNB to guide adjuvant treatment.^21,22^ Understanding the patterns of adherence to existing guidelines which are restricted to older patients and their implications for patient care may help guide future management of an even larger and more inclusive population of women diagnosed with this disease.

We had hypothesized that as the use of SLNB declined in this population post-CW, we would also see a gradual decline in the utilization of postoperative therapies as consistent with the overall trend of treatment de-escalation. Instead, while rates of postoperative chemotherapy were consistent, we observed a significant increase in the use of postoperative radiation amongst patients who did not undergo SLNB. Previous authors have compared the rates of adjuvant radiation between SLN positive and negative patients since the publication of the CW guidelines and have shown that nodal positivity is associated with increased likelihood of receiving radiation.^6,8^ In addition, one prior study looked specifically at the rates of radiation between those who did or did not receive SLNB and found that receipt of biopsy itself was associated with increased adjuvant radiation.^6^ Our study is consistent with these findings, but to our knowledge is the first to consider how management of patients in whom SLNB is omitted is changing over time after publication of the CW guidelines. The reason for the increased rates of radiation experienced within this group remains unknown, though it may reflect provider hesitancy to de-escalate treatment in the setting of less information regarding pathologic nodal staging.^23,24^ Nodal status may be particularly pertinent for decision-making regarding adjuvant radiation with patients undergoing mastectomy, and while those patients were found to be independently more likely to undergo SLNB in the post-CW cohort, a significant majority (85.7%) of the cohort did not undergo SLNB. The interdisciplinary nature of cancer care poses unique challenges for overall treatment de-escalation and was identified as an important factor in a qualitative study assessing lack of adherence to CW recommendations.^18^ That study suggested that preoperative conversations between medical, surgical, and radiation oncologists may mitigate some of the concerns regarding both omission of SLNB and adjuvant radiation.

Our study has some important notable limitations. First, though we adjusted our multivariable models for measured confounders, our results remain impacted by unmeasured confounding. While the NCDB captures data from more than 1500 medical centers, it is limited to those accredited by the Commission-on-Cancer, potentially limiting the generalizability of our results. In addition, while we excluded patients with missing data with the assumption that the data is missing at random, there may have been unequal distribution of missing baseline characteristics and outcomes across treatment groups, thereby confounding our findings. Details about chemotherapy regimens and radiation treatments are also lacking in the database, although presumably there would not be significant variability in these treatments in this clinical setting. Finally, while we were able to examine trends in practice patterns and thereby make inferences about guideline adherence, the database lacks information about provider-level decision-making.

## Conclusions

This study shows that there has been significant progress made in de-escalating surgical management of early-stage breast cancer in older patients, although a majority of patients are still undergoing SLNB. Moreover, we found that patients in whom SLNB is being omitted appear more likely than before to receive adjuvant radiation. Continuing to focus on both patient and surgeon education, emphasizing the evidence behind the recommendations, as well as the overall excellent prognosis associated with early-stage disease remains essential to providing quality uniform and cost-effective care for all patients.
